# Novel OBP genes similar to hamster Aphrodisin in the bank vole, *Myodes glareolus*

**DOI:** 10.1186/1471-2164-11-45

**Published:** 2010-01-19

**Authors:** Romana Stopková, Zbyněk Zdráhal, Štěpán Ryba, Ondřej Šedo, Martin Šandera, Pavel Stopka

**Affiliations:** 1 Department of Zoology, Faculty of Science, Charles University in Prague, Viničná 7, Prague, CZ-128 44, Czech Republic; 2 Department of Functional Genomics and Proteomics, Institute of Experimental Biology, Faculty of Science, Masaryk University, Brno, Kamenice 5 (A2), Brno, CZ-625 00, Czech Republic

## Abstract

**Background:**

Chemical communication in mammals involves globular lipocalins that protect and transport pheromones during their passage out of the body. Efficient communication via this protein - pheromone complex is essential for triggering multiple responses including aggression, mate choice, copulatory behaviour, and onset and synchronization of oestrus. The roles of lipocalins in communication were studied in many organisms and especially in mice (i.e. *Mus musculus domesticus*) which excrete Major Urinary Proteins (Mup) in excessive amounts in saliva and urine. Other mammals, however, often lack the genes for Mups or their expression is very low. Therefore, we aimed at characterization of candidate lipocalins in *Myodes glareolus *which are potentially linked to chemical communication. One of them is Aphrodisin which is a unique lipocalin that was previously described from hamster vaginal discharge and is known to carry pheromones stimulating copulatory behaviour in males.

**Results:**

Here we show that Aphrodisin-like proteins exist in other species, belong to a group of Odorant Binding Proteins (Obp), and contrary to the expression of Aphrodisin only in hamster genital tract and parotid glands of females, we have detected these transcripts in both sexes of *M. glareolus *with the expression confirmed in various tissues including prostate, prepucial and salivary glands, liver and uterus. On the level of mRNA, we have detected three different gene variants. To assess their relevance for chemical communication we investigated the occurrence of particular proteins in saliva, urine and vaginal discharge. On the protein level we confirmed the presence of Obp2 and Obp3 in both saliva and urine. Appropriate bands in the range of 17-20 kDa from vaginal discharge were, however, beyond the MS detection limits.

**Conclusion:**

Our results demonstrate that three novel Obps (Obp1, Obp2, and Obp3) are predominant lipocalins in *Myodes *urine and saliva. On the protein level we have detected further variants and thus we assume that similarly as Major Urinary Proteins in mice, these proteins may be important in chemical communication in this *Cricetid *rodent.

## Background

Lipocalins are known to be involved in many important biological processes, of which one of them is chemical communication. Due to a conservative tertiary structure forming typical beta barrel all lipocalins share ability to bind various ligands of different size and structure. For some of the lipocalin members there already exists evidence for causing significant behavioural effects by their specific pheromonal ligands. The main lipocalin proteins studied to date in association with chemical communication are the mouse Major Urinary Proteins (Mup) secreted predominantly in the urine and saliva and Odorant Binding Proteins (Obp) with predominant site of expression in nasal tissue.

In mice, urine is an important source of chemical information, frequently investigated by conspecifics and having the ability to elicit various behavioural responses including aggression, dominance and mate choice [1-6]. The major components of mouse urine are the Major Urinary Proteins, constituting approximately 90% of all proteins in the urine. The amount of Mups excreted to urine is sexually dimorphic, with males expressing more Mups than females on both mRNA and protein level [7]. However even the lower amount of Mups in female urine may be sufficient as a signal of reproductive status, advertising the beginning of oestrus [8]. Various substances with pheromonal effect bound to Major Urinary Proteins were identified [3,9-11] and many of studies have brought the evidence that these Mup-ligand complexes [12-14], unbounded ligands and potentially Mup itself [4,12] have effect upon the reproductive physiology of the signal/pheromone receiver.

Mups are coded by high number of genes which have probably evolved by recent gene duplication and form a cluster of 21 intact genes and 21 pseudogenes on chromosome 4 [15,16]. Such variability leads to the hypothesis that urine marks may serve for individual odour discrimination in mice [17]. However, high variability of Mups is clearly demonstrated only in particular subspecies of the house mouse *Mus musculus domesticus *and derived laboratory strains and is completely missing in some other rodent species.

Contrary to Mup sexual dimorphism biased to males there exists another lipocalin in hamsters, called Aphrodisin, of which the expression is attributed only to females. Aphrodisin is a lipocalin that was isolated from hamster vaginal discharge by Singer et al. [18] and is detected (or it's ligands) via the vomeronasal organ of male hamsters. The term Aphrodisin originates from its functional property to stimulate copulatory behaviour in male hamsters which were exposed to vaginal odour of females [19]. Analysis of various fractions of female vaginal discharge led to the conclusion that this lipocalin itself is responsible for various behavioural and physiological effects [18]. However later studies revealed that recombinant Aphrodisin produced in *E. coli *was clearly less active than Aphrodisin isolated from vaginal discharge [20]. It, however, remains to be determined whether 3D structure of lipocalins produced in bacteria corresponds to the structure detected in mammals that naturally produce aphrodisin. The effect of Aphrodisin may be enhanced by various ligands that were detected in hamster vaginal smears by Briand et al. [21] who described five specific compounds which are also present in insect pheromone blends. Analysis of the Aphrodisin expression sites revealed that the main tissue expressing this gene is vagina with the Bartholin's glands [22-24], cervix uteri, low level of expression was detected also in ovaries [23], and moreover in the parotid glands of females [24]. Expression of Aphrodisin is attributed only to females, nevertheless there is weak evidence for the absence of Aphrodisin in males, because there is only one paper [24] from available literature where the researchers analyzed male saliva and parotid glands and had found no expression. To our knowledge, there is as yet no published information on the expression of Aphrodisin in male bulbourethral glands which are homologous to female Bartholin's glands.

According to the protein sequence analysis and of exon-intron organization, hamster Aphrodisin falls within the monophyletic group of lipocalins (described as cluster X) including i.a. mouse Obps, rat Obp1f and Probasin [25,26]. Protein sequence comparison of Aphrodisin shows the highest similarity with the mouse Obp1a and Obp1b (~50%) [24] and ~40% similarity with the rat Obp1f [21]. Our presented novel sequences on the translated protein level share 59% similarity with this hamster lipocalin. In mice Odorant Binding Protein forms heterodimer containing 18 kDa and 19 kDa subunits called Obp1a and Obp1b, which are expressed mainly in the main olfactory epithelium [27]. In our previous paper [16] we have analyzed public databases (NCBI) and proposed that the mouse Obp forms a gene cluster of more than only two Obp variants. On the X chromosome in the range of 0.5 Mb there are at least 8 Obp genes, including one pseudogene and these genes can be further divided into three structural groups [16]. Lipocalins linked to the monophyletic cluster X [25], including all mouse Obp genes, homologous rat Obp genes from X chromosome and Aphrodisin share typical sequence characterized by extra two cystein residues forming CxxxC motif [16]. In addition to the conservative disulfide bond tightening the beta-barel to the C-terminal tail [28] this extra two cystein residues form a second disulfide bridge characteristic for this group of lipocalins [29]. Interestingly, this motif also occurs in some other lipocalin genes, namely Probasin, with chromosomal location next to the Obp cluster, and recently described hamster's Male Specific Salivary Protein (MSP) and Female Lacrimal Protein (FLP) [30-32]. It suggests that despite the different names, these lipocalins belong to the Obp group and may also act as odorant/pheromone carriers, however, this has to be further studied.

Obps are known to bind various odorant molecules [33] and are found through the wide spectrum of animals [27,34-38]. The suggested role of Obp in olfaction predicts the transport of hydrophobic odorants across the aqueous mucous layer to the odorant receptors [39-41], termination of olfactory signals by removing the odorants from the receptor [35] or enhancing the signal by maintain the active conformation of olfactory receptors and thus contribute to signal amplification [42,43]. Boudjelal et al. [44] pointed out that the receptor for Obp is presented in number of tissues unrelated to olfaction and therefore suggested that the function of this lipocalin may not be limited only to odorant transport. Another feature of lipocalins is their ability to scavenge for toxic substances or act as an antibacterial defence. The scavenger function of porcine and bovine Obp was suggested [45] in an experiment which clearly showed that Obp binds with high affinity HNE (4-hydroxy-2-nonenal), a toxic compound derived from lipid peroxidation, and therefore can protect living cells from damage caused by oxidative stress by removing cytotoxic compounds from the nasal mucus. The antibacterial defence arises from the ability of some lipocalins (i.e. called siderocalins) to bind bacterial siderophores and thus prevent the bacterial growth [46-48]. There are few candidate siderocalins in mammals (Lcn1, Lcn2 and Lcn12), however it is possible that due to the conservative tertiary structure some other lipocalins may also share this ability [49].

The main goal of this paper was to identify dominant transcripts/proteins in *Myodes glareolus *that are secreted to urine, saliva and vaginal secrets and propose potential roles for these proteins. Bank voles are a target species in various ecological studies and our previous analysis revealed that individuals of both sex contain candidate proteins of approximately 17-20 kDa in the urine. However, there is little available information on the mechanisms that bank voles use to mark their territories, attract individuals of opposite sex and how is this information transmitted to other individuals. Therefore, this study employed gene expression and gene characterization techniques to uncover a potential group of proteins that may play a role in chemical communication in this species.

## Results

### *M. glareolus *voles express three novel Obp genes/transcripts in different secretory tissues

**Figure 1:**
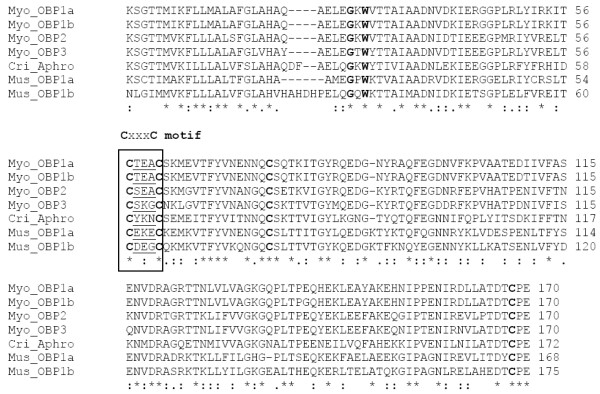
**Alignment of translated novel sequences from *Myodes glareolus***. Myo_Obp1a, Myo_Obp1b, Myo_Obp2 and Myo_Obp3 are compared with known lipocalins of the mouse (Obp1a: Mus_Obp1a and Obp1b: Mus_Obp1b) and hamster (Aphrodisin: Cri_Aphro). Typical CxxxC motif is visualized by a box.

RT-PCR was conducted using mRNA from secretory tissues (prostate, preputial glands, salivary glands, liver). The samples were derived from wild caught *M. glareolus *to ascertain whether *M. glareolus *express transcripts for candidate lipocalins that are known to carry volatiles facilitating chemical communication. Use of a hamster Aphrodisin specific primer set generated clear bands in the expected area. *M. glareolus *amplicons were purified and cloned with TA TOPO kit and consequently the total of 28 clones were each forward and reverse sequenced. This analysis revealed three unique cDNA amplicons described as Obp1 [GQ219783], Obp2 [GQ219784], and Obp3 [GQ219785] (Figure 1) which were similar to hamster Aphrodisin (75% identity) and mouse Obp1a and Obp1b (72% identity) from public NCBI database. Sequencing further revealed that 20 sequences - identified as Obp1 - were present in individuals of both sex and across all tissues analyzed except male prostate. Three nucleotide polymorphisms were revealed in two of the female samples (i.e. liver, parotid glands) causing 1% difference (i.e. 99% identity) from Obp1 for which we use the term Obp1b. However, this could be a result of heterozygous effect. The Obp2 transcripts were detected in male prostate (3 clones, i.e. 6 sequences) whilst Obp3 was detected from a single clone using cDNA from submandibular glands.

**Figure 2:**
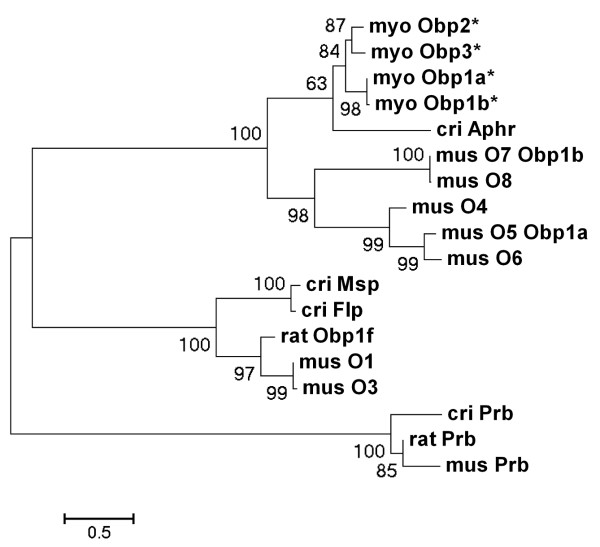
**The evolutionary history of Obp using the Neighbour-Joining method**. Novel sequences of *Myodes glareolus *are marked with * and described as myo Obp1-3. Mouse Obp sequences are depicted as mus O1-O8 assigned with respective synonyms Obp1a and Obp1b. Known lipocalin sequences from *Cricetus cricetus *are Aphrodisin (cri Aphr), Male salivary protein (cri Msp), Female lacrimal protein (cri Flp) and Probasin (cri Prb). In *Rattus norvegicus *the only two lipocalins belonging to this cluster are Obp1f (rat Obp1f) and Probasin (rat Prb). The optimal tree with the sum of branch length = 11.92163796 is shown. The percentage of replicate trees in which the associated taxa clustered together in the bootstrap test (1000 replicates) are shown next to the branches. The tree is drawn to scale, with branch lengths in the same units as those of the evolutionary distances used to infer the phylogenetic tree. The evolutionary distances were computed using the Maximum Composite Likelihood method and are in the units of the number of base substitutions per site. All positions containing gaps and missing data were eliminated from the dataset (Complete deletion option). There were a total of 478 positions in the final dataset. Phylogenetic analyses and this caption were conducted in MEGA4 software [57].

More detailed analysis of translated transcripts revealed that the novel Obp genes have typical lipocalin signatures including GxW motif [50], two conservative cystein residues characteristic for most of the lipocalins, and extra two cysteins forming the CxxxC motif (see Figure 1). This motif is a specific feature of hamster Aphrodisin described as a position of the second disulphide bond in this lipocalin [29]. To add, all mouse Obps, Rat Obp1f, and other lipocalins compared in Figure 2 share the same feature.

Contrary to hamsters, the expression of novel genes is not female specific but is present in individuals of both sex and across different tissues studied. Although, these novel sequences were highly similar to hamster Aphrodisin (75% identity) we use more general term Obps (i.e. Obp stands for Odorant Binding Protein) with which they share 72% identity (Figure 2) because the term Aphrodisin presupposes its aphrodisiac function which was not assessed for novel proteins in this study.

### 2-D electrophoresis and MS analyses confirmed the presence of detected Obps in the urine and saliva but not in vaginal flushes

**Figure 3:**
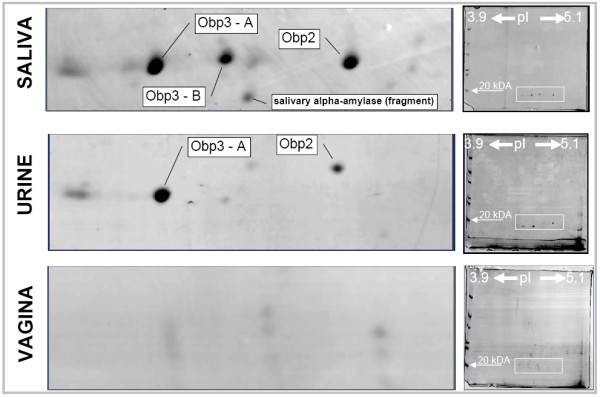
**Results 2-DE analysis confirmed the presence of Obp proteins in the urine and saliva**. All visible spots have been analyzed with MS/MS techniques where the presence of Obps was detected in most of them. However, only annotated spots (i.e. Obp2, Obp3-A, Obp3-B) were reliably assigned to the particular sequences depicted in Figure 1 and Figure 4.

**Figure 4:**
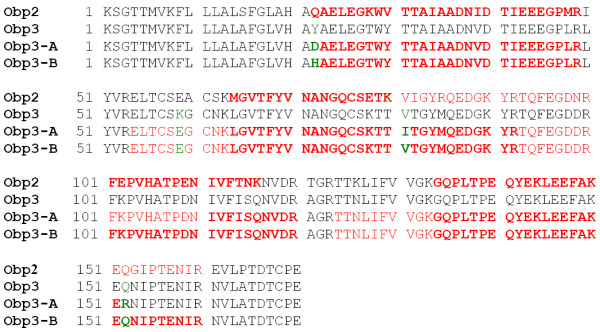
**Comparison of Obp sequences identified in *M. glareolus *salivary samples**. Sequence regions elucidated by mass spectrometry are marked in color. Regions in red bold were confirmed by both MS/MS techniques, regions in red normal were confirmed by one of the employed MS/MS techniques. Sequence positions in green bold correspond to amino acid changes in Obp3 variants. The regions in black are derived only from the clone nucleotide sequences and were not confirmed by mass spectrometry.

Image analysis of 2D gels of urinary, salivary and vaginal samples revealed protein spots in the predicted region of pI (3.9-5.1) and in the MW range of 18-20 kDa. After tryptic digestion protein spots were subjected to MALDI-MS/MS and LC-MS/MS analysis. MS/MS data were primarily searched against to the Obp library containing DNA- derived sequences. Subsequently, the data were searched against NCBI protein database to avoid random false positives caused by small size of the Obp library. MS/MS data of peptides which have not matched to Obp library in Mascot database search were manually evaluated. Based on MS/MS data three Obp proteins were unambiguously identified: Obp2 and two Obp3 isoforms (Obp3-A and Obp3-B). These proteins correspond to the most intensive spots in 2D gel of the salivary sample. Only Obp2 and Obp3-A were identified in the urinary sample (see Figure 3). The transcript corresponding to Obp1 gene was abundant on the level of mRNA, however, remained undetected on the level of protein. Reasons for this failure may involve modifications that changed its pI or that the concentration of Obp1 protein was low and thus below MS identification limits. Furthermore, the Obp proteins were present also in less intense spots but their identification was based on sequence regions identical for all variants not allowing correct determination of the particular variant and they are not further discussed in this study. Results of MS/MS analysis of three main Obp variants in salivary samples are summarized in Figure 4.

Sequence coverage ranged from 63% (Obp2) to 77% (Obp3-B) when non-specific trypsin cleavage were taken in account (enzyme "semiTrypsin"). While detected parts of Obp2 sequence were completely in agreement with cDNA-derived sequence, Obp3 protein sequence showed several differences from cDNA-derived sequence indicating the presence of two Obp3 variants (see Figure 4). The amino acid changes were tentatively identified and their confirmation on the mRNA and protein level is a subject of ongoing projects. Complete results of Mascot searches against Obp sequences library including amino acid corrections for Obp3 are available in Additional file 1.

## Discussion

Wild caught *M. glareolus *voles transcribe (and translate) three novel Obp genes highly similar to those previously described as hamster Aphrodisin. Exon 2, encoding the first sheet of beta barrel, includes a specific signature in amino acid sequence (GxW) that places these novel genes to a family of proteins called lipocalins. As in mouse and hamster these *Myodes *Obp genes also include specific amino acid feature CxxxC which is known to form disulphide bond in hamster Aphrodisin. For its ability to form a pheromone-carrying beta barrel this protein is important as it may have overtaken the role of Major Urinary Proteins (Mups) in those species where Mups are not present or are expressed in low quantities. This argument is further supported by studies that show Mups being important in individual recognition and mate choice [17,51]. If lipocalins are generally important in these processes they must by substituted by a similar sort of proteins in those species where the expression of Mups is lost.

*M. glareolus *Obp (i.e. Obp1, Obp2, Obp3) mRNA derived nucleotide sequences (cDNA) were aligned against the corresponding consensus sequences from the *Mus, Rattus*, and *Cricetus *sequences from NCBI library. The analyses of replicate trees in which the associated taxa/proteins clustered together revealed that novel *Myodes *Obps cluster together with *Cricetus *Aphrodisin, thus, forming a parallel cluster to mouse Obp1a, Obp1b and remaining *Mus *Obps (bootstrap = 100) previously described as O4-O8 in Stopková et al. [16]. To add, both *Cricetus *Aphrodisin and *Myodes *Obp form a group of proteins that, due to a high structural similarity may also serve similar functions where a capacity to bind and transport pheromones by *Cricetus *Aphrodisin has already been documented [21,52]. Furthermore, the method of clustering in Figure 2 shows that the divergence of Obp-like lipocalins caused by gene duplication preceded a mouse - rat split that occurred 10.3 millions of years ago as well as muroid and cricetid divergence that most likely occurred 22.5 millions years ago [53]. This is seen in Prb (Probasin) with a branching pattern where a mouse - rat split was preceded by a split of the mouse - rat common ancestor from *Cricetid *rodents (i.e. *Cricetus, Myodes etc*.).

In contrast to mouse Major Urinary Proteins with 21 genes and 21 pseudogenes, Obps in *Myodes glareolus *were detected as only three unique transcripts. However, our 2-DE and consequent MS/MS analyses indicate that the number of potential protein products depicted in Figure 3 may be higher and that further analysis is required to uncover other potential genes and/or allelic variants. On the level of proteins the number of detected variants exceeded the number of detected transcripts in case of Obp3 (Figure 4) and several other Obp proteins were detected in low abundant spots.

To add *Myodes glareolus *Obp is highly similar to *Cricetus *and *Mesocricetus *Aphrodisin, however, their expression pattern is different. Contrary to hamsters, *M. gareolus *Obp was detected in both sexes and in all tested tissues whilst it was missing in vaginal flushes or its expression was low. Its absence might have been caused by the fact that the main site of Obp production in this area are prepucial glands which secret this protein in to the vaginal lumen under specific stimuli which our experimental sample collection method did not satisfy.

## Conclusions

*Myodes glareolus *voles express three novel Aphrodisin-like genes here named as Obp1, Obp2, and Obp3. Proteomic analysis further revealed that there may be further variants of this genes and that they are secreted into urine and saliva. The comparative analysis provided evidence that these transcripts are expressed in all studied tissues and by individuals of both sex. All this evidence - along with the fact that almost identical Obps in other species have binding affinity to pheromones - suggests that these proteins may be used in *M. glareolus *for chemical communication similarly as Major Urinary Proteins in mice.

## Methods

### Subjects and samples

The total of twenty individual bank voles (10 males and 10 females, *Myodes glareolus*) were caught in the Czech Republic in Cernovice near Tabor, near Prague and near Prachov City using the Chmela wild traps. All animals were individually caged with food and water provided *ad libitum*. All conditions of the Czech law were met to diminish animal suffering under captive rearing. Urine samples were collected in the first half of the 14:10 light period by spontaneous lavage straight to eppendorf tubes, whilst salivary and vaginal samples were collected by gentle flushes with a 10 μl Eppendorf tips and with the total volume of 50 μl of distilled water.

### Analysis of transcripts

At the day of tissue extraction animals were sacrificed by cervical dislocation and five tissue samples were collected from each animal. Tissue samples were obtained from prepucial/clitorial glands, prostate, liver, parotid glands, submandibular glands and whole uterus tract (without ovaries). Immediately after resection each tissue sample was placed into eppendorff tube with mixture of Trizol (TRIzol Reagent - Invitrogen) and glass pellets and homogenized using mill homogenizator (MM200 - Retsch). RNA was isolated using standard Trizol protocol and followed by cDNA synthesis using First strand cDNA synthesis kit (Fermentas).

Various primer sets were tested to amplify the "Aphrodisin" gene, derived from known sequence of hamster Aphrodisin (*Mesocricetus auratus, Cricetus cricetus*). Finally we set up one pair of primers giving one clear band in the expected area. This primer set covers the whole region from start to stop codon (i.e. Forward: CAAGTCAGGCACCACCATG and Reverse: TTTATTCAGGACAAGTATCTG). Using following conditions for PCR reaction (denaturation 95°C for 30s, annealing 56°C for 30s and extension 75°C for 50s, repeated for 35 cycles) we get in all tissues one clear band in the expected range of 515 bp checked in agarose gel. These PCR products from all tissues were purified using QIAquick PCR Purification Kit (Qiagen). We have cloned the PCR product of two males and two females from different tissues separately. Cloning into pCRII TA vector was made as described by the manufacturer (TOPO TA Cloning Kit - Dual promoter, Invitrogen). Clones were sequenced (3130 Genetic Analyzer, Applied Biosystems) using a forward and reverse M13 primers. Sequences were analyzed using Sequence Scanner (Applied Biosystem) software and compared with known sequences from public database NCBI using BLAST. Novel sequences were deposited in GeneBank with the accession numbers provided in the results section.

### Protein electrophoresis

The Bradford method [54] was used to detect appropriate concentration of proteins for further analysis. The total of 5 μl (urine), 5 μl (saliva), or 11.25 μl (vaginal flush) of sample was acetone precipitated. The pellet was diluted in 10 μl of SDS sample buffer and heated at 70°C for 10 minutes. Separation was performed by means of denaturing polyacrylamide gel electrophoresis (SDS-PAGE) using 10% NuPAGE^® ^Novex Bis-Tris Midi Gels and NuPAGE^® ^MES Running Buffer (Invitrogen Technologies, Paisley, UK). The gels were stained with the Colloidal Coomassie^® ^G-250 (SimplyBlue™ Safe Stain, Invitrogen Life Technologies, Paisley, UK) with images being acquired by GS-800 Calibrated Densitometer.

Two dimensional polyacrylamide elctrophoresis (2D-PAGE) was performed with IEF cell (Bio-rad^®^) and Protean II electrophoresis system. For the first dimension 12 μg of proteins was applied to Bio-Rad 17 cm strips (pI: 3.9-5.1). Isoelectric focusing was performed after passive rehydration at room temperature and run at 50 V 9.5 hrs, 250 V (rapid) for 15 min, 500 V (rapid), 1000 V (rapid) for 15 min, 10000 V (rapid) - 35000 V/hrs, and finished at 500 V until further step. For the second- dimension separation, the strips were equilibrated for 10 minutes in 45 mM Tris base (pH 7.0) containing 6 M urea, 1.6% SDS, 30% glycerol, and 130 mM dithiothreitol, and then re-equilibrated for 10 minutes in the same buffer containing 135 mM iodoacetamide in place of dithiothreitol. The strips were then placed on Protean II hand-made 12% gels, and unstained molecular standards were applied. Second dimension gels were run at constant current - 50 mA for 1 hr, 100 mA for 1 hr and 150 mA for 1.5 hrs at 10°C. After electrophoresis, the gels were stained with the Colloidal Coomassie^® ^G-250 (SimplyBlue™ Safe Stain, Invitrogen Life Technologies, Paisley, UK) with images being acquired by GS-800 Calibrated Densitometer. All spots in the range 15-25 kDa were excised with a Bio-rad Spot Cutter.

### Mass Spectrometric Analysis

Protein spots selected for analysis were excised from 2-DE gels. After destaining, the proteins in gel pieces were incubated with trypsin (sequencing grade, Promega) at 37°C for 2 h. Digested peptides were extracted from gels using 50% ACN solution with 5% formic acid.

MALDI-MS and MS/MS analyses were performed on an Ultraflex III mass spectrometer (Bruker Daltonik, Bremen, Germany). Peptide maps were acquired in reflectron positive mode (25 kV acceleration voltage) with 800 laser shots. Peaks within 700 - 4000 Da mass range and minimum S/N 10 were picked out for MS/MS analysis employing LID-LIFT arrangement with 600 laser shots for each peptide. CHCA solution prepared according to Havlis [55] was used as the matrix in combination with AnchorChip target to enhance measurement sensitivity. Sample (1 μl) was mixed with matrix solution on the target in a 2:1 ratio. Known autoproteolytic products of trypsin were used for internal calibration of digested peptides. In the absence of these products, an external calibration procedure was employed, using a mixture of seven peptide standards (Bruker Daltonik) covering the mass range of 1000-3100 Da. The Flex Analysis 3.0 and MS Biotools 3.1 (Bruker Daltonik) software were used for data processing.

LC-MS/MS experiments were accomplished on an HPLC system consisting of a gradient pump (Ultimate), autosampler (Famos) and column switching device (Switchos; LC Packings, Amsterdam, The Netherlands) on-line coupled with an HCTultra PTM Discovery System ion trap mass spectrometer (Bruker Daltonik). Tryptic digests were concentrated and desalted using PepMap C18 trapping column (300 μm × 5 mm, LC Packings). Sample volume was 15 μl. After washing with 0.1% formic acid, the peptides were eluted from the trapping column using an acetonitrile/water gradient (4 μL/min) onto a fused-silica capillary column (320 μm × 180 mm), on which peptides were separated. This column was filled with 4-μm Jupiter Proteo sorbent (Phenomenex, Torrance, CA) according to a previously described procedure [56]. The mobile phase A consisted of acetonitrile/0.1% formic acid (5/95 v/v) mixture and the mobile phase B consisted of acetonitrile/0.1% formic acid (80/20 v/v) mixture. The gradient elution started at 5% of mobile phase B, and after 4 minutes, it was increased linearly from 5% to 50% during 55 minutes. The analytical column outlet was connected to the electrospray ion source via a 50-μm-inner diameter fused-silica capillary. Nitrogen was used as nebulizing as well as drying gas. The pressure of nebulizing gas was 15 psi. The temperature and flow rate of drying gas were set to 300°C and 6 L/min, respectively, and the capillary voltage was 4.0 kV. The mass spectrometer was operated in the positive ion mode in a *m/z *range of 300 - 1500 for MS and 100-3000 for MS/MS scans. Extraction of the mass spectra from the chromatograms, mass annotation and deconvolution of the mass spectra were performed using DataAnalysis 4.0 software (Bruker Daltonik).

### Data Processing

MASCOT 2.2 (MatrixScience, London, UK) search engine was used for processing the MS and MS/MS data. Database searches were done against the NCBI protein database (Release 20090210) as well as against translated sequence data obtained by TOPO cloning experiment. A mass tolerance of 60 ppm was allowed during processing MALDI MS data for PMF and 0.7 Da during processing LID-LIFT data for MS/MS ion searches. For ESI-MS/MS data, mass tolerances of peptides and MS/MS fragments for MS/MS ion searches were 0.5 Da. All searches were done without taxonomic restriction. Both trypsin and semitrypsin as an enzyme were selected for each sample. Oxidation (M), carbamidomethylation (C), deamidation (N, Q) and pyro-Glu (Q, N-term) as optional modifications and up to three enzyme miscleavages were set for all searches. Unassigned MS/MS spectra were subjected to manual interpretation to elucidate peptide primary structure and its changes from given sequence data.

## Authors' contributions

RS designed primers screened different individuals and tissues, sequenced all transcripts and prepared the draft of this manuscript. ZZ and OS performed all MS analysis including tryptic digestions and consequent analysis of MS/MS spectra. ŠR performed clonning. MŠ coordinated the sacrificing and with RS performed disections and collected all the mRNA and protein samples. PS coordinated the project, prepared protein gels (2-DE) and helped with the manuscript. All authors have read and approved the final manuscript.

## Supplementary Material

Additional file 1 **Final Results of Mascot MS/MS Ion Search**. The file contains the list of peptides identified by Mascot MS/MS Ion Search for particular Obps. Click here for file
