# Supplementary material for: Novel OBP genes similar to hamster Aphrodisin in the bank vole, Myodes glareolus
Source: BMC Genomics. 2010 Jan 19;11:45. doi: 10.1186/1471-2164-11-45 (PMC2824723; doi:10.1186/1471-2164-11-45)
Supplement: Additional file 1 — Final Results of Mascot MS/MS Ion Search. The file contains the list of peptides identified by Mascot MS/MS Ion Search for particular Obps. [file 1471-2164-11-45-S1.pdf]

Final Results of Mascot MS/MS Ion Search

Search was done against library of DNA derived Obp sequences. Obp3-A and Obp3-B sequences contained amino acid corrections proposed by manual processing of MS/MS data
Limit value score was 26

Salivary sample

Obp2
MALDI-MS/MS

Seq. region	Observed	Mr(expt)	Mr(calc)	ppm	Miss	Score	Expect	Rank	Peptide
81 - 92	1483.79	1482.78	1482.76	14	2	35	0.0028	1	K.VIGYRQEDGKYR.T
64 - 80	1921.84	1920.83	1920.83	-2	0	138	1.3e-013	1	K.MGVTFYVNANGQCSETK.V + Carbamidomethyl (C); Oxidation (M)
134 - 150	2007.00	2006.00	2006.00	-2	1	62	7.3e-006	1	K.GQPLTPEQYEKLEEFAK.E
29 - 49	2332.12	2331.11	2331.11	3	0	185	5.4e-018	1	K.WVTTAIAADNIDTIEEEGPMR.I
64 - 85	2494.19	2493.18	2493.18	1	1	77	3.2e-007	1	K.MGVTFYVNANGQCSETKVIGYR.Q + Carbamidomethyl (C)
93 - 116	2790.34	2789.33	2789.34	-5	1	74	7.4e-007	1	R.TQFEGDNRFEPVHATPENIVFTNK.N
22 - 49	3070.45	3069.45	3069.46	-5	1	256	5.3e-025	1	A.QAELEGKWVTTAIAADNIDTIEEEGPMR.I + Gln->pyro-Glu (N-term Q)
134 - 160	3144.57	3143.57	3143.58	-4	2	203	1.1e-019	1	K.GQPLTPEQYEKLEEFAKEQGIPTENIR.E

LC-MS/MS

Seq. region	Observed	Mr(expt)	Mr(calc)	Delta	Miss	Score	Expect	Rank	Peptide
22 - 28	379.2	756.3	756.4	-0.1	0	28	0.054	1	A.QAELEGK.W + Gln->pyro-Glu (N-term Q)
127 - 133	388.2	774.5	774.5	0.0	0	31	0.031	1	K.LIFVVGK.G
145 - 160	625.3	1872.8	1873.0	-0.1	1	55	0.00015	1	K.LEEFAKEQGIPTENIR.E
64 - 80	641.6	1921.7	1921.8	-0.1	0	86	8.3e-008	1	K.MGVTFYVNANGQCSETK.V + Carbamidomethyl (C); Deamidated (NQ); Oxidation (M)
37 - 49	759.7	1517.4	1517.7	-0.3	0	41	0.0036	1	A.DNIDTIEEEGPMR.I
68 - 80	759.8	1517.5	1517.6	-0.2	0	45	0.0014	1	T.FYVNANGQCSETK.V + Carbamidomethyl (C); Deamidated (NQ)
34 - 49	887.3	1772.6	1772.8	-0.2	0	82	3.8e-007	1	A.IAADNIDTIEEEGPMR.I
136 - 150	911.4	1820.7	1820.9	-0.2	1	60	5.9e-005	1	Q.PLTPEQYEKLEEFAK.E
101 - 116	921.9	1841.7	1841.9	-0.2	0	41	0.0034	1	R.FEPVHATPENIVFTNK.N
33 - 49	922.8	1843.6	1843.9	-0.3	0	67	9.4e-006	1	T.AIAADNIDTIEEEGPMR.I

Obp3 - A
MALDI-MS/MS

Seq. region	Observed	Mr(expt)	Mr(calc)	ppm	Miss	Score	Expect	Rank	Peptide
79 - 92	1662.79	1661.78	1661.77	7	1	115	3.4e-012	1	K.TTITGYMQEDGKYR.T
134 - 152	2292.16	2291.15	2291.14	5	2	139	1.3e-014	1	K.GQPLTPEQYEKLEEFAKER.N
54 - 78	2836.25	2835.24	2835.26	-6	1	239	1.3e-024	1	R.ELTCSEGCNKLGVTFYVNANGQCSK.T + 3 Carbamidomethyl (C)
22 - 49	3079.42	3078.41	3078.43	-6	0	318	1.5e-032	1	K.DAELEGTWYTTAIAADNVDTIEEEGPLR.L
93 - 120	3245.57	3244.57	3244.59	-7	1	101	8.8e-011	1	R.TQFEGDDRFKPVHATPDNIVFISQNVDR.A
124 - 152	3364.75	3363.75	3363.77	-8	3	277	2.2e-028	1	R.TTNLIFVVGKGQPLTPEQYEKLEEFAKER.N

LC-MS/MS

Seq. region	Observed	Mr(expt)	Mr(calc)	Delta	Miss	Score	Expect	Rank	Peptide
125 - 133	495.8	989.5	989.6	-0.1	0	45	0.0012	1	T.TNLIFVVGK.G
124 - 133	546.3	1090.5	1090.6	-0.1	0	56	4.5e-005	1	R.TTNLIFVVGK.G
111 - 120	595.8	1189.5	1189.6	-0.1	0	27	0.043	1	N.IVFISQNVDR.A
22 - 32	643.2	1284.4	1284.6	-0.1	0	59	2.8e-005	1	K.DAELEGTWYTT.A
134 - 144	645.3	1288.5	1288.6	-0.1	0	37	0.0041	1	K.GQPLTPEQYEK.L
79 - 90	672.2	1342.4	1342.6	-0.2	0	31	0.016	1	K.TTITGYMQEDGK.Y
22 - 33	678.7	1355.5	1355.6	-0.1	0	62	1.3e-005	1	K.DAELEGTWYTTA.I
82 - 92	682.2	1362.4	1362.6	-0.2	1	66	5.2e-006	1	I.TGYMQEDGKYR.T + Oxidation (M)
38 - 49	686.3	1370.6	1370.7	-0.1	0	49	0.00025	1	D.NVDTIEEEGPLR.L
136 - 152	702.9	2105.8	2106.1	-0.3	2	61	1.9e-005	1	Q.PLTPEQYEKLEEFAKER.N
81 - 92	730.8	1459.5	1459.7	-0.2	1	76	5.6e-007	1	T.ITGYMQEDGKYR.T

37 - 49	743.8	1485.5	1485.7	-0.2	0	90	2.3e-008	1	A.DNVDTIEEEGPLR.L
134 - 146	766.3	1530.6	1530.8	-0.1	1	31	0.018	1	K.GQPLTPEQYEKLE.E
64 - 78	830.3	1658.5	1658.8	-0.2	0	96	6.1e-009	1	K.LGVTFYVNANGQCSK.T + Carbamidomethyl (C); 2 Deamidated (NQ)
79 - 92	831.8	1661.6	1661.8	-0.2	1	110	2.3e-010	1	K.TTITGYMQEDGKYR.T
34 - 49	871.3	1740.7	1740.9	-0.2	0	94	8.7e-009	1	A.IAADNVDTIEEEGPLR.L
33 - 49	906.8	1811.6	1811.9	-0.3	0	103	1.1e-009	1	T.AIAADNVDTIEEEGPLR.L
136 - 150	911.3	1820.7	1820.9	-0.2	1	85	7.5e-008	1	Q.PLTPEQYEKLEEFAK.E
134 - 150	1003.9	2005.8	2006.0	-0.2	1	72	1.6e-006	1	K.GQPLTPEQYEKLEEFAK.E

Obp3 - B

MALDI-MS/MS

Seq. region	Observed	Mr(expt)	Mr(calc)	ppm	Miss	Score	Expect	Rank	Peptide
79 - 92	1648.73	1647.72	1647.76	-20	1	81	8.1e-009	1	K.TTVTGYMQEDGKYR.T
134 - 150	2006.95	2005.95	2006.00	-27	1	74	4.2e-008	1	K.GQPLTPEQYEKLEEFAK.E
101 - 120	2297.14	2296.13	2296.20	-28	0	52	6.5e-006	1	R.FKPVHATPDNIVFISQNVDR.A
54 - 78	2836.10	2835.15	2835.26	-40	1	102	6.4e-011	1	R.ELTCSEGCNKLGVTFYVNANGQCSK.T + 3 Carbamidomethyl (C)
22 - 49	3101.35	3100.34	3100.46	-38	0	227	3.7e-023	1	K.HAELEGTWYTTAIAADNVDTIEEEGPLR.L
134 - 160	3201.49	3200.48	3200.64	-48	2	179	1.6e-018	1	K.GQPLTPEQYEKLEEFAKEQNIPTENIR.N
93 - 120	3245.48	3244.47	3244.59	-38	1	145	4.1e-015	1	R.TQFEGDDRFKPVHATPDNIVFISQNVDR.A

LC-MS/MS

Seq. region	Observed	Mr(expt)	Mr(calc)	Delta	Miss	Score	Expect	Rank	Peptide
124 - 133	546.3	1090.5	1090.6	-0.1	0	58	2.6e-005	1	R.TTNLIFVVGK.G
22 - 31	603.7	1205.4	1205.5	-0.2	0	30	0.016	1	K.HAELEGTWYT.T
151 - 160	607.2	1212.5	1212.6	-0.2	0	52	9.3e-005	1	K.EQNIPTENIR.N
68 - 78	644.7	1287.4	1287.6	-0.1	0	62	1.3e-005	1	T.FYVNANGQCSK.T + Carbamidomethyl (C); Deamidated (NQ)
134 - 144	645.2	1288.5	1288.6	-0.2	0	45	0.00076	1	K.GQPLTPEQYEK.L
22 - 32	654.2	1306.4	1306.6	-0.1	0	42	0.001	1	K.HAELEGTWYTT.A
79 - 90	665.2	1328.4	1328.6	-0.1	0	76	4.8e-007	1	K.TTVTGYMQEDGK.Y
82 - 92	682.2	1362.4	1362.6	-0.1	1	50	0.00019	1	V.TGYMQEDGKYR.T + Oxidation (M)
38 - 49	686.2	1370.5	1370.7	-0.2	0	48	0.00029	1	D.NVDTIEEEGPLR.L
22 - 33	689.7	1377.5	1377.6	-0.1	0	55	5.3e-005	1	K.HAELEGTWYTTA.I
81 - 92	723.7	1445.5	1445.7	-0.2	1	55	5.6e-005	1	T.VTGYMQEDGKYR.T
37 - 49	743.8	1485.5	1485.7	-0.2	0	97	4.3e-009	1	A.DNVDTIEEEGPLR.L
101 - 120	766.3	2295.9	2296.2	-0.3	0	62	1.6e-005	1	R.FKPVHATPDNIVFISQNVDR.A
36 - 49	779.3	1556.6	1556.7	-0.2	0	76	4.4e-007	1	A.ADNVDTIEEEGPLR.L
79 - 92	824.8	1647.6	1647.8	-0.2	1	90	2.6e-008	1	K.TTVTGYMQEDGKYR.T
64 - 78	830.3	1658.6	1658.8	-0.2	0	78	3.1e-007	1	K.LGVTFYVNANGQCSK.T + Carbamidomethyl (C); 2 Deamidated (NQ)
34 - 49	871.3	1740.6	1740.9	-0.2	0	99	2.5e-009	1	A.IAADNVDTIEEEGPLR.L
33 - 49	906.9	1811.7	1811.9	-0.2	0	107	3.7e-010	1	T.AIAADNVDTIEEEGPLR.L
136 - 150	911.4	1820.7	1820.9	-0.2	1	70	2.1e-006	1	Q.PLTPEQYEKLEEFAK.E
145 - 160	965.9	1929.8	1930.0	-0.2	1	49	0.00027	1	K.LEEFAKEQNIPTENIR.N
134 - 150	1003.9	2005.8	2006.0	-0.2	1	48	0.00033	1	K.GQPLTPEQYEKLEEFAK.E

Urinary sample

Obp2

MALDI-MS/MS

Seq. region	Observed	Mr(expt)	Mr(calc)	ppm	Miss	Score	Expect	Rank	Peptide

81 - 92	1483.78	1482.77	1482.76	10	2	64	3.5e-006	1	K.VIGYRQEDGKYR.T
64 - 80	1921.84	1920.84	1920.83	1	0	127	1.8e-012	1	K.MGVTFYVNANGQCSETK.V + Carbamidomethyl (C); Oxidation (M)
134 - 150	2007.01	2006.00	2006.00	1	1	91	9.8e-009	1	K.GQPLTPEQYEKLEEFAK.E
29 - 49	2348.10	2347.10	2347.10	-2	0	78	2.1e-007	1	K.WVTTAIAADNIDTIEEEGPMR.I + Oxidation (M)
93 - 116	2790.34	2789.33	2789.34	-4	1	104	7.6e-010	1	R.TQFEGDNRFEPVHATPENIVFTNK.N
22 - 49	3086.42	3085.41	3085.46	-13	1	90	2.3e-008	1	A.QAELEGKWVTTAIAADNIDTIEEEGPMR.I + Gln->pyro-Glu (N-term Q); Oxidation (M)
134 - 160	3144.56	3143.55	3143.58	-9	2	243	1.3e-023	1	K.GQPLTPEQYEKLEEFAKEQGIPTENIR.E
93 - 120	3274.53	3273.53	3273.58	-16	2	36	0.0051	1	R.TQFEGDNRFEPVHATPENIVFTNKNVDR.T
86 - 116	3666.69	3665.69	3665.75	-17	3	35	0.007	1	R.QEDGKYRTQFEGDNRFEPVHATPENIVFTNK.N

LC-MS/MS

Seq. region	Observed	Mr(expt)	Mr(calc)	Delta	Miss	Score	Expect	Rank	Peptide
127 - 133	388.2	774.4	774.5	-0.1	0	55	5.7e-005	1	K.LIFVVGK.G
151 - 160	578.8	1155.5	1155.6	-0.1	0	34	0.0077	1	K.EQGIPTENIR.E
134 - 144	645.3	1288.5	1288.6	-0.1	0	35	0.0077	1	K.GQPLTPEQYEK.L
103 - 116	783.8	1565.7	1565.8	-0.2	0	31	0.015	1	E.PVHATPENIVFTNK.N
34 - 49	895.3	1788.6	1788.8	-0.2	0	103	9.1e-010	1	A.IAADNIDTIEEEGPMR.I + Oxidation (M)
136 - 150	911.4	1820.7	1820.9	-0.2	1	74	9.4e-007	1	Q.PLTPEQYEKLEEFAK.E
101 - 116	921.9	1841.7	1841.9	-0.2	0	62	1.4e-005	1	R.FEPVHATPENIVFTNK.N
33 - 49	930.8	1859.7	1859.9	-0.2	0	77	5.6e-007	1	T.AIAADNIDTIEEEGPMR.I + Oxidation (M)
145 - 160	937.4	1872.8	1873.0	-0.2	1	63	1.1e-005	1	K.LEEFAKEQGIPTENIR.E
64 - 80	961.8	1921.6	1921.8	-0.2	0	109	1.8e-010	1	K.MGVTFYVNANGQCSETK.V + Carbamidomethyl (C); Deamidated (NQ); Oxidation (M)

Obp3 - A

MALDI-MS/MS

Seq. region	Observed	Mr(expt)	Mr(calc)	ppm	Miss	Score	Expect	Rank	Peptide
79 - 92	1678.79	1677.78	1677.77	8	1	63	5.3e-007	1	K.TTITGYMQEDGKYR.T + Oxidation (M)
134 - 150	2007.01	2006.00	2006.00	2	1	76	2.3e-008	1	K.GQPLTPEQYEKLEEFAK.E
54 - 78	2837.26	2836.25	2836.25	2	1	204	3.7e-021	1	R.ELTCSEGCNKLGVTFYVNANGQCSK.T + 3 Carbamidomethyl (C); Deamidated (NQ)
22 - 49	3079.43	3078.43	3078.43	-1	0	266	2.7e-027	1	K.DAELEGTWYTTAIAADNVDTIEEEGPLR.L
93 - 120	3245.57	3244.56	3244.59	-9	1	87	2.6e-009	1	R.TQFEGDDRFKPVHATPDNIVFISQNVDR.A

LC-MS/MS

Seq. region	Observed	Mr(expt)	Mr(calc)	Delta	Miss	Score	Expect	Rank	Peptide
124 - 133	546.3	1090.5	1090.6	-0.1	0	63	9.1e-006	1	R.TTNLIFVVGK.G
39 - 49	629.3	1256.5	1256.6	-0.1	0	28	0.037	1	N.VDTIEEEGPLR.L
83 - 92	631.6	1261.3	1261.5	-0.3	1	34	0.016	1	T.GYMQEDGKYR.T + Oxidation (M)
22 - 32	643.2	1284.4	1284.6	-0.1	0	76	6.2e-007	1	K.DAELEGTWYTT.A
134 - 144	645.3	1288.5	1288.6	-0.1	0	30	0.024	1	K.GQPLTPEQYEK.L
22 - 33	678.7	1355.5	1355.6	-0.1	0	82	1.5e-007	1	K.DAELEGTWYTTA.I
82 - 92	682.2	1362.4	1362.6	-0.2	1	54	9.1e-005	1	I.TGYMQEDGKYR.T + Oxidation (M)
38 - 49	686.3	1370.5	1370.7	-0.1	0	72	1.4e-006	1	D.NVDTIEEEGPLR.L
136 - 152	703.0	2105.8	2106.1	-0.2	2	36	0.0058	1	Q.PLTPEQYEKLEEFAKER.N
81 - 92	738.8	1475.5	1475.7	-0.2	1	56	6.5e-005	1	T.ITGYMQEDGKYR.T + Oxidation (M)
37 - 49	743.8	1485.5	1485.7	-0.2	0	75	7.6e-007	1	A.DNVDTIEEEGPLR.L
101 - 120	766.3	2295.9	2296.2	-0.3	0	55	0.00016	1	R.FKPVHATPDNIVFISQNVDR.A
36 - 49	779.3	1556.6	1556.7	-0.2	0	80	2.2e-007	1	A.ADNVDTIEEEGPLR.L
35 - 49	814.8	1627.5	1627.8	-0.2	0	64	8.1e-006	1	I.AADNVDTIEEEGPLR.L
64 - 78	830.3	1658.6	1658.8	-0.2	0	110	2.6e-010	1	K.LGVTFYVNANGQCSK.T + Carbamidomethyl (C); 2 Deamidated (NQ)
79 - 92	839.8	1677.5	1677.8	-0.2	1	70	2.5e-006	1	K.TTITGYMQEDGKYR.T + Oxidation (M)
34 - 49	871.3	1740.6	1740.9	-0.3	0	99	3.1e-009	1	A.IAADNVDTIEEEGPLR.L
33 - 49	906.8	1811.6	1811.9	-0.2	0	107	4.9e-010	1	T.AIAADNVDTIEEEGPLR.L
136 - 150	911.3	1820.6	1820.9	-0.3	1	88	3.6e-008	1	Q.PLTPEQYEKLEEFAK.E
134 - 150	1003.9	2005.7	2006.0	-0.3	1	76	6.2e-007	1	K.GQPLTPEQYEKLEEFAK.E
